# Aujeszky’s Disease and the Development of the Marker/DIVA Vaccination Concept

**DOI:** 10.3390/pathogens9070563

**Published:** 2020-07-12

**Authors:** Thomas C. Mettenleiter

**Affiliations:** Friedrich-Loeffler-Institut, Federal Research Institute for Animal Health, Südufer 10, 17493 Greifswald-Insel Riems, Germany; thomas.mettenleiter@fli.de

**Keywords:** pseudorabies, Aujeszky’s disease, Aladár Aujeszky, pseudorabies virus, marker vaccine, DIVA strategy

## Abstract

Aujeszky’s disease or pseudorabies is an infection of animals caused by Suid alphaherpesvirus 1, also designated as pseudorabies virus (PrV). Whereas many mammals are susceptible to PrV, only pigs are able to survive productive infection. Early reports on this disease originate from cattle and companion animals with the hallmark sign of “mad itch”, meaning development of pruritus. Although first reports date back to the early 19th century, it was Aladár Aujeszky who in 1902 described this disease, which has since been named after him, as a separate entity. AD expanded in the 20th century, despite efforts to control this infection in the growing pig farming industry. Live-attenuated vaccines were developed in the early 1960s, which assisted early eradication efforts. A major breakthrough in animal vaccinology occurred in the mid-1980s, when it was found that several live-attenuated PrV vaccine strains lacked a significant portion of the genome, including the gene encoding a major immunogenic viral envelope glycoprotein. Upon the development of a suitable serological assay, the first marker vaccine/DIVA concept (differentiating infected from vaccinated animals) was developed. Moreover, the first genetically modified live vaccines emanated from molecular work on PrV. Thus, AD serves as a hallmark for the history of veterinary virology as well as for pioneering novel strategies for controlling animal infectious diseases.

## 1. Early Reports on “Mad Itch”

The causative agent of Aujeszky’s disease (AD) is an alphaherpesvirus species now taxonomically correctly designated as *Suid Alphaherpesvirus 1* in the genus *Varicellovirus* of the Subfamily *Alphaherpesvirinae* of the Family *Herpesviridae* in the Order *Herpesvirales*. Although the taxonomic species name indicates that the natural host of pseudorabies virus (PrV) is pigs, clinical symptoms of PrV were first described in 1813 in cattle. This was because PrV infection in swine, particularly in older animals, may produce only innocuous respiratory signs or may be clinically inapparent, whereas productive infection in other susceptible species is invariably fatal and characterized by severe central nervous signs. Thus, the rabies-like clinical picture in cattle prompted the use of the term “pseudorabies” in Switzerland in 1849 [1]. Likewise, “mad itch” was used to describe the disease in cattle in the United States in the first half of the 19th century because PrV causes excessive pruritus [2]. The term “mad itch” first appeared in 1839 and 1844 in two farmer’s journals in the US, but was described by its clinical picture already in 1823 [2]. In Switzerland, in 1889, a similar “mad itch” disease (in German: ‘Juckkrankheit’) was described in cattle; this clinical picture had been seen before in cattle and dogs [3]. Thus, by the end of the 19th century the disease was already well recognized but there was no information on the causative agent.

## 2. Aladár Aujeszky and ‘Aujeszky’s Disease’

**Figure 1:**
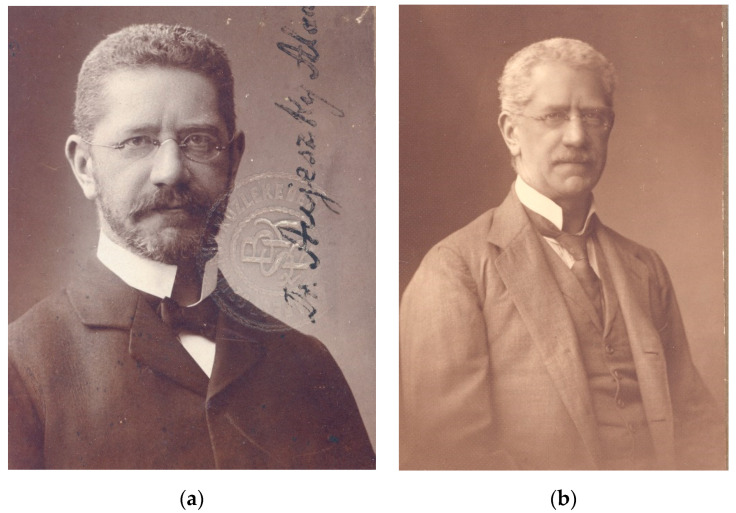
Aladár Aujeszky at younger (**a**) and senior age (**b**). Pictures by courtesy of Hutӱra Ferenc Library, Archives and Museum, University of Veterinary Science, Budapest, Hungary.

**Figure 2:**
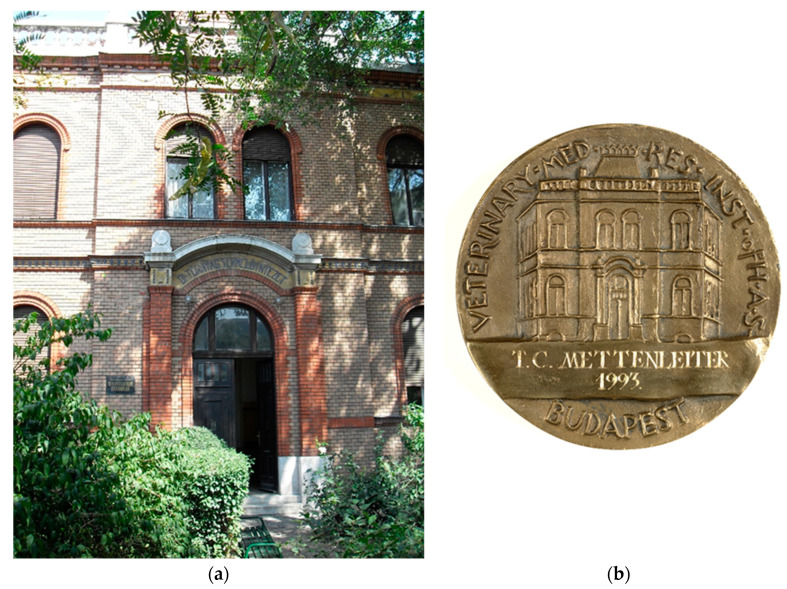
Former Hungarian Royal Bacteriological Institute and present Institute for Veterinary Medical Research of the Hungarian Academy of Sciences (**a**), pictured also on reverse side of the Aladár Aujeszky memorial medal (**b**).

The Hungarian physician Aladár Aujeszky (Figure 1) was the first to scientifically describe the disease that bears his name. He was born in Budapest on 11 January 1869, and graduated from the the Hungarian Royal Medical University in Budapest in 1892. Thereafter, he worked in practice for three years, but in 1895 went to the Pasteur Institute in Paris to gain experience in bacteriology. After his return to Hungary, in 1896 he received a position in the General Pathological Institute of the Medical University as an Assistant Professor. With a priority interest in infectious diseases after his stay at the Pasteur Institute, he focused on pathogenesis, pathology, and etiology of human infectious diseases. In 1901, he joined the Hungarian Royal Bacteriological Institute to work on rabies. This institute was part of the Veterinary College under the auspices of the Ministry of Agriculture [4]. The building still exists today (Figure 2). Interestingly, in this environment medical and veterinary microbiologists worked together in a context that we would now call “One Medicine/One Health”. Working in an institution with a priority on rabies research and considering his training at the Pasteur Institute, Aujeszky studied cases of putatively “rabid” animals, in particular livestock. 

In 1902, he reported the isolation of the infectious agent from a diseased ox, a dog, and a cat, and differentiated it from rabies [5]. He concluded “The described disease is in some characteristics strictly different from rabies, and its virus is not identical with rabies virus” [5]. It could be passaged in rabbits reproducing the typical clinical signs. Guinea pigs and mice were also found to be susceptible, whereas chickens and doves were resistant. Subsequently, the disease pseudo-rabies became widely known as Aujeszky’s disease (AD). Interestingly, in his report he used the term “infectious virus” to describe the agent, which was impossible to culture and to detect with bacteriological methods. Surprisingly, early filtration experiments gave mixed results as to whether the infectious agent of AD was indeed a “filterable virus” [3]. This was finally proven by filtration experiments through Berkefeld and Chamberland filters by Shope [6] and Elford and Galloway [7]. The latter estimated that the particle size would be ca. 100-150 nm, which is remarkably close to the 180 nm estimate of today. 

**Figure 3:**
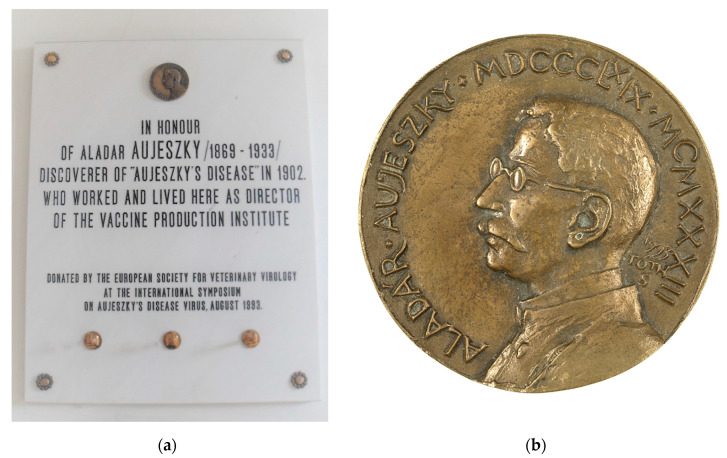
Memorial plaque donated by the European Society for Veterinary Virology located in the entrance hallway of the institute (**a**) and Aladár Aujeszky memorial medal (**b**) established by the Veterinary Research Institute of the Hungarian Academy of Sciences.

In 1903, Aladár Aujeszky received a veterinary degree from the college and became director of the Bacteriological Institute in 1906. From 1907 until his death, he was involved in vaccine production and control in Hungary. He was instrumental in eradicating dog-mediated rabies in Hungary by the 1930′s. Aladár Aujeszky died on March 9, 1933 in Budapest. In 1993, the 60th anniversary of his death, the European Society for Veterinary Virology sponsored a memorial plaque, which is mounted in the hallway of the institute (Figure 3a). The memorial lecture was given by Prof. Adorjan Bartha [4]. In parallel, the Veterinary Research Institute of the Hungarian Academy of Sciences established an Aladár Aujeszky memorial medal (Figure 3b), which was presented to several scientists working on AD for their achievements. 

It was not until 1931 that Richard Shope established that the agent of “mad itch” was also present in domestic pig holdings in the United States [6]. Erich Traub was the first to cultivate PrV in vitro in organ explants in 1933 [8]. He performed research on Insel Riems in Germany, at Princeton University (Princeton, NJ, USA) and at Plum Island (Southold, NY, USA), and thereafter became the first president of the West German “Federal Research Centre for Virus Diseases of Animals” in Tübingen. In 1934, Sabin and Wright reported a serological relationship between PrV and herpes simplex virus, resulting in the inclusion of PrV into the herpesvirus group [9]. Finally, in 1959 Kaplan and Vatter directly compared herpes simplex 1 and pseudorabies viruses and proved their close relationship [10]. 

## 3. Pseudorabies (Aujeszky’s Disease) in Pigs

Whereas PrV exhibits a wide host range capable of infecting basically all mammals except higher primates and equids, only pigs are able to survive a productive infection and are thus considered the natural host [11]. AD in German pig holdings was first described in 1941 [12], but has been present in US American holdings and several European countries beyond Hungary since the early 20th century. PrV infections in swine soared after the Second World War, particularly in Europe, when intensive pig breeding and farrowing were established. In the 1970s, PrV became a major scourge of pigs worldwide, distributed primarily by global movement of animals and animal products.

Although field isolates and strains differ in virulence, they can cause devastating losses by fatal infection of piglets and abortions in pregnant animals. Pigs exhibit a pronounced age resistance against PrV, with younger animals being more susceptible to fatal infections characterized by neuronal signs, such as ataxia, convulsions, and sudden death. In contrast, older animals (>1 year) primarily present with respiratory distress or even subclinical infection. In pregnant animals, infection of fetuses results in resorption, mummification, or abortion [13].

Pigs are the only natural host for PrV, but the virus can naturally also infect cattle, sheep, cats, dogs, mice, and rats, causing fatal disease [14]. Infections have also been reported in brown bear, black bear, Florida panther, raccoon, coyote, deer, and farm fur animal species (mink and foxes). Recently, isolated transient infections in humans have been reported, although a body of evidence indicates humans are highly resistant to infection (see review by Sehl and Teifke in this issue [12,15]). Only swine (Suidae) are able to survive a productive PrV infection [14]. Of the laboratory species, rabbits are most susceptible and develop intense local pruritus at the inoculation site. Guinea pigs are less susceptible and may resist subcutaneous inoculation, but succumb to intracerebral or intraperitoneal inoculation [16].

Due to the rapid increase in AD in the 1970s, test and slaughter programs were initiated in several countries, including England, Switzerland, and Denmark, in the early 1980s [17]. Although costly, they succeeded in eliminating AD from national pig herds, but new outbreaks occurred due to introduction of virus by trade or air. In other countries, control of disease but not infection was achieved by blanket vaccination with inactivated (particularly in breeding animals) and modified live virus vaccines (in finishers). In East Germany, for example, AD control was based on a combination of large-scale vaccination of breeding herds with attenuated live vaccines and a rigorous stamping-out policy, especially in the final phase of eradication until successful termination of the AD eradication program in 1985 [18]. Whereas inactivated and attenuated vaccines were efficacious in reducing disease, they did not lead to the elimination of virus, since none of them prevented latent infection and subsequent reactivation and shedding of virulent field virus. 

## 4. Novel Strategies for Animal Disease Control: Marker Vaccines and DIVA Tests

Beginning in the 1980s, novel strategies in animal disease control were pioneered by the first use of genetically engineered live PrV vaccines lacking virulence-determining genes (reviewed in [19]). In 1986, the first recombinant DNA-derived modified live virus vaccine was licensed in the United States [20,21]. It carried a genetically engineered deletion of the gene encoding thymidine kinase, a protein that was known to be relevant for virulence. At about the same time, it was discovered that several classical live-attenuated AD vaccine strains, for example, the Bartha “K” strain [22,23]), carried large deletions in the unique short portion of their genomes [24], including the gene encoding immunogenic virion envelope glycoprotein gE (previously designated gI) [25]. Absence of this major but nonessential glycoprotein from the deleted strains was verified [26], but its absence apparently did not impair their potency as vaccines. After the development of a serological test (ELISA) to detect anti-gE antibodies in the animal [27], the combination of marker vaccine and differential ELISA made it possible to discriminate between vaccinated, PrV-noninfected animals (PrV-positive but anti-gE-negative) from wild-type PrV-infected (anti-gE-positive) animals. Subsequently, other nonessential glycoproteins, for example, gC or gG, were also deleted by genetic engineering and used as markers with appropriate serological assay systems (reviewed in [19]). Since then, AD has been the prime and highly successful example of using marker vaccines in combination with companion diagnostic ELISA tests to differentiate infected from vaccinated animals (DIVA) [19]. Thus, marker vaccines against AD were the first genetically modified live vaccines used on a wide scale in Europe [28]. Detailed reviews of the development of PrV vaccines have been published elsewhere [19,29,30]. The combination of highly efficacious marker vaccines and accurate differential ELISAs has made eradication of AD from large areas of the world by a DIVA strategy [31] practical and feasible. 

Due to its excellent safety and efficacy profile, PrV live-attenuated vaccines have also been developed into viral vectors, allowing immunization against AD as well as heterologous viral infections by expression, e.g., of glycoprotein E2 of classical swine fever virus [32] or swine influenza hemagglutinin [33].

## 5. Conclusions

In summary, PrV is certainly the best-studied herpesvirus of animals in terms of its importance in the field, eradication strategies, vaccine development, and molecular biology [34]. Although eradication has been achieved in several countries, AD in pigs remains a challenge, in particular in South America, Africa, and Asia, which justifies ongoing research to improve control efforts and to understand in detail all facets of this “queen of herpesviruses” (Quote: Lynn W. Enquist, Princeton, NY, USA).

## References

[1] Wyssmann E. (1942). Nochmals zur Frage des Vorkommens der Aujeszky’schen Krankheit, Pseudowut, in der Schweiz. Schweiz. Arch. Tierheilkd..

[2] Hansen R.P. (1954). The history of pseudorabies in the United States. J. Am. Vet. Med. Assoc..

[3] Köhler M., Köhler W. (2003). Zentralblatt für Bakteriologie-100 years ago: Aladár Aujeszky detects a ‘new’ disease–or: It was the cow and not the sow. Int. J. Med. Microbiol..

[4] Bartha A. (1994). Aujeszky Memorial Lecture. Acta Vet. Hung..

[5] Aujeszky A. (1902). Ueber eine neue Infektionskrankheit bei Haustieren. Cbl. Bakteriol Infektionskrankh. Parasitenkde. 1 Abt. Orig..

[6] Shope R.E. (1931). An experimental study of ‘mad itch’ with especial reference to its relationship to pseudorabies. J. Exp. Med..

[7] Elford W.J., Galloway I.A. (1936). The Size of the Virus of Aujeszky’s Disease (“Pseudo-rabies”, “Infectious Bulbar Paralysis”, “Mad-itch”) by Ultrafiltration Analysis. J. Hyg..

[8] Traub E. (1933). Cultivation of pseudorabies virus. J. Exp. Med..

[9] Sabin A.B., Wright A.M. (1934). Acute ascending myelitis following a monkey bite, with the isoltion of a virus capable of reproducting the disease. J. Exp. Med..

[10] Kaplan A.S., Vatter A.E. (1959). A comparison of herpes simplex and pseudorabies viruses. Virology.

[11] Kretzschmar C. (1970). Die Aujeszkysche Krankheit.

[12] Heynen K. (1941). Untersuchungen über den Nachweis und das Vorkommen der Pseudowut (Aujeszkyschen Krankheit) bei Schweinen in Deutschland. Z. Infekt.-Krankh. Parasitären Krankh. u. Hyg. d. Haustiere.

[13] Mettenleiter T.C., Ehlers B., Müller T., Yoon K.-J., Teifke J.P., Zimmerman J.J., Karriker L.A., Ramirez A., Schwartz K.J., Stevenson G.W., Zhang J. (2019). Herpesviruses. Diseases of Swine.

[14] Pensaert M., Kluge P., Pensaert M. (1989). Pseudorabies virus (Aujeszky’s disease). Virus Infections of Porcines.

[15] Sehl J., Teifke J.P. (2020). Comparative pathology of pseudorabies in different naturally and experimentally infected species—A review. Pathogens.

[16] Ashworth L.A., Baskerville A., Lloyd G. (1980). Aujeszky’s disease in the guinea pig: Cellular and humoral responses following immunization. Arch. Virol..

[17] Watson W.A. (1986). Epidemiology and control of Aujeszky’s disease in Great Britain. Rev. Sci. Tech..

[18] Müller T., Bätza H.J., Schlüter H., Conraths F.J., Mettenleiter T.C. (2003). Eradication of Aujeszky’s disease in Germany. J. Vet. Med. B Infect. Dis. Vet. Public Health.

[19] Freuling C.M., Muller T.F., Mettenleiter T.C. (2017). Vaccines against pseudorabies virus (PrV). Vet. Microbiol..

[20] Kit S., Kit M., Pirtle E.C. (1985). Attenuated properties of thymidine kinase-negative deletion mutant of pseudorabies virus. Am. J. Vet. Res..

[21] Kit S., Sheppard M., Ichimura H., Kit M. (1987). Second-generation pseudorabies virus vaccine with deletions in thymidine kinase and glycoprotein genes. Am. J. Vet. Res..

[22] Bartha A. (1961). Attempts at reduction of virulence of Aujeszky virus. Mag. Allator. Lap..

[23] Bartha A. (1962). Immunization experiments with the attenuated, K’strain of Aujeszky virus. Mag. Allator. Lap..

[24] Lomniczi B., Blankenship M.L., Ben-Porat T. (1984). Deletions in the genomes of pseudorabies virus vaccine strains and existence of four isomers of the genomes. J. Virol..

[25] Mettenleiter T.C., Lukacs N., Rziha H.J. (1985). Mapping of the structural gene of pseudorabies virus glycoprotein A and identification of two non-glycosylated precursor polypeptides. J. Virol..

[26] Mettenleiter T.C., Lukacs N., Rziha H.J. (1985). Pseudorabies virus avirulent strains fail to express a major glycoprotein. J. Virol..

[27] van Oirschot J.T., Rziha H.J., Moonen P.J., Pol J.M., van Zaane D. (1986). Differentiation of serum antibodies from pigs vaccinated or infected with Aujeszky’s disease virus by a competitive enzyme immunoassay. J. Gen. Virol..

[28] Quint W., Gielkens A., Vanoirschot J., Berns A., Cuypers H.T. (1987). Construction and Characterization of Deletion Mutants of Pseudorabies Virus-a New Generation of Live Vaccines. J. Gen. Virol..

[29] Mengeling W.L., Brockmeier S.L., Lager K.M., Vorwald A.C. (1997). The role of biotechnologically engineered vaccines and diagnostics in pseudorabies (Aujeszky’s disease) eradication strategies. Vet. Microbiol..

[30] Dong B., Zarlenga D.S., Ren X. (2014). An overview of live attenuated recombinant pseudorabies viruses for use as novel vaccines. J. Immunol. Res..

[31] van Oirschot J.T. (1999). Diva vaccines that reduce virus transmission. J. Biotechnol..

[32] van Zijl M., Wensvoort G., de Kluyver E., Hulst M., van der Gulden H., Gielkens A., Berns A., Moormann R. (1991). Live attenuated pseudorabies virus expressing envelope glycoprotein E1 of hog cholera virus protects swine against both pseudorabies and hog cholera. J. Virol..

[33] Klingbeil K., Lange E., Teifke J.P., Mettenleiter T.C., Fuchs W. (2014). Immunization of pigs with an attenuated pseudorabies virus recombinant expressing the haemagglutinin of pandemic swine origin H1N1 influenza A virus. J. Exp. Med..

[34] Pomeranz L.E., Reynolds A.E., Hengartner C.J. (2005). Molecular biology of pseudorabies virus: Impact on neurovirology and veterinary medicine. Microbiol. Mol. Biol. Rev..

